# Supplementary material for: Causal interplay between lactose intolerance and gut microbiota: a combined bidirectional Mendelian randomization and in vivo validation study
Source: Front Nutr. 2026 Jun 1;13:1803337. doi: 10.3389/fnut.2026.1803337 (PMC13265576; doi:10.3389/fnut.2026.1803337)
Supplement: Supplementary file 1 [file Data_Sheet_1.zip › supplementary materials/Forward/leave one out cross validation/ebi-a-GCST90027506.finngen_R12_E4_LACTONAS.pdf]

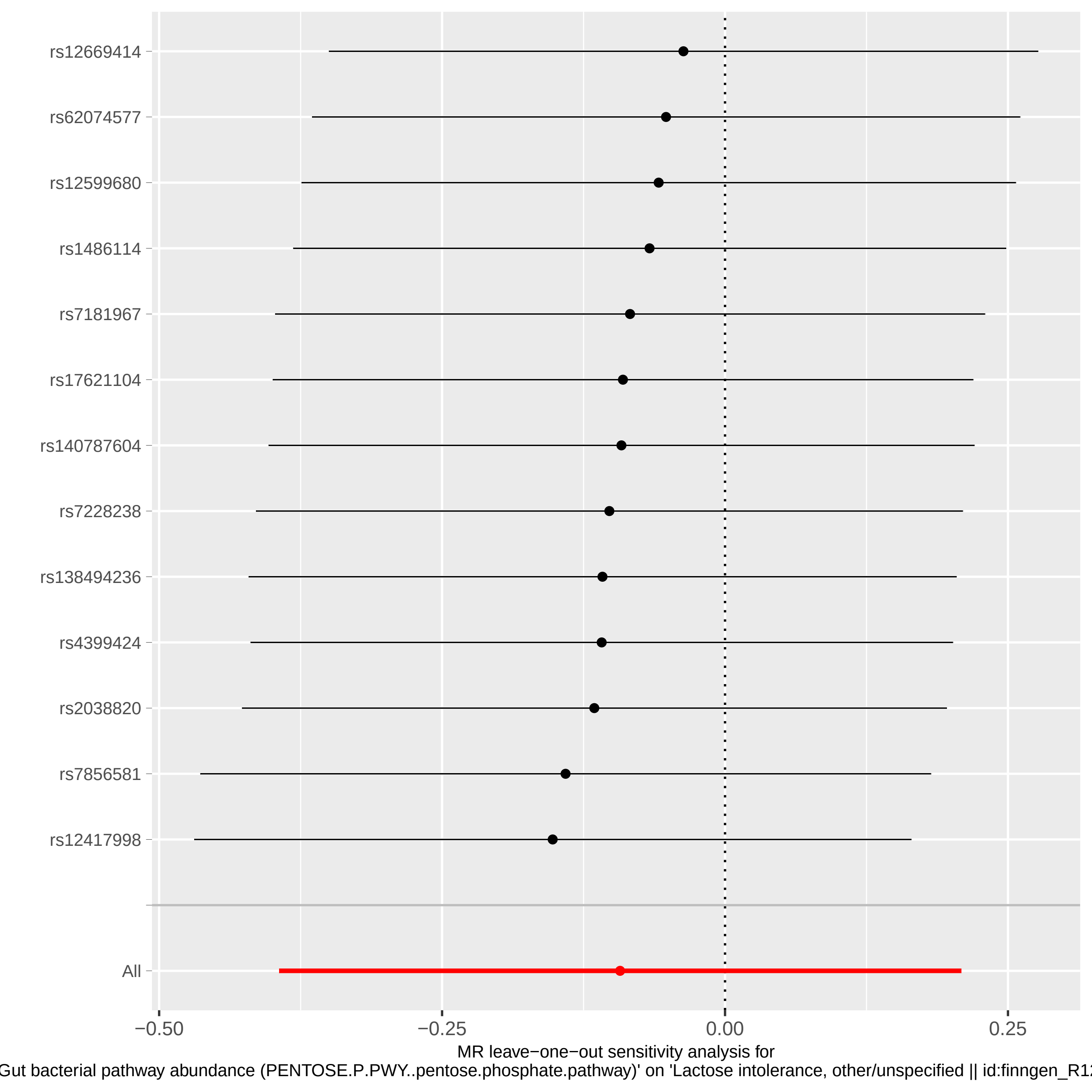

rs12669414
rs62074577
rs12599680
rs1486114
rs7181967
rs17621104
rs140787604
rs7228238
rs138494236
rs4399424
rs2038820
rs7856581
rs12417998
All
−0.50
−0.25
0.00
0.25
MR leave−one−out sensitivity analysis for
Gut bacterial pathway abundance (PENTOSE.P.PWY..pentose.phosphate.pathway)' on 'Lactose intolerance, other/unspecified || id:finngen_R1
